# 1,2-Bis{2-[(4-meth­oxy­benzyl­idene)amino]­phen­yl}disulfane

**DOI:** 10.1107/S1600536813011598

**Published:** 2013-05-15

**Authors:** Wei Xin

**Affiliations:** a Kingfa Scientific & Technological Coporation Limited, Guang Zhou, China 510663, People’s Republic of China

## Abstract

The asymmetric unit of the title compound, C_28_H_24_N_2_O_2_S_2_, contains one-half mol­ecule, which is completed by twofold rotation symmetry with the twofold axis passing through the mid-point of the central S—S bond. The planes of the two benzene rings linked by the di­sulfide chain form a dihedral angle of 76.1 (1)°, while the planes of the two benzene rings in the benzyl­ideneaniline fragment form a dihedral angle of 48.9 (1)°. The crystal packing exhibits no significantly short inter­molecular contacts.

## Related literature

For the crystal structures of related compounds, see: İde *et al.* (1997 ▶); Ozbey *et al.* (1998 ▶); He *et al.* (2011 ▶); Wang *et al.* (2011 ▶).
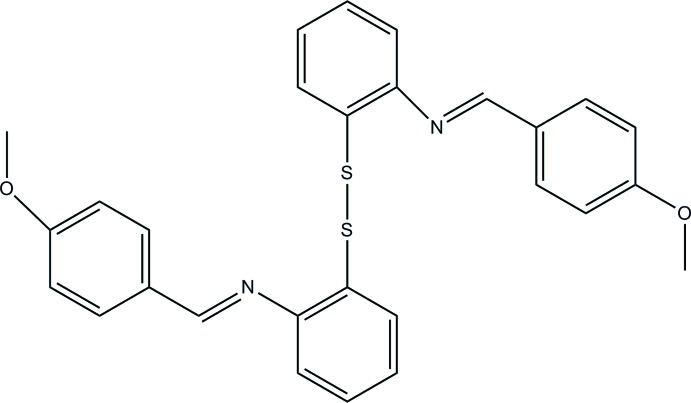


## Experimental

### 

#### Crystal data

C_28_H_24_N_2_O_2_S_2_
*M* *_r_* = 484.61
Orthorhombic, 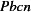

*a* = 10.2657 (11) Å
*b* = 13.0675 (13) Å
*c* = 18.3415 (15) Å
*V* = 2460.5 (4) Å^3^
*Z* = 4
Mo *K*α radiation
μ = 0.25 mm^−1^
*T* = 298 K
0.26 × 0.22 × 0.17 mm

#### Data collection

Bruker SMART APEX CCD area-etector diffractometer
Absorption correction: multi-scan (*SADABS*; Sheldrick, 1996 ▶) *T*_min_ = 0.939, *T*_max_ = 0.960
9487 measured reflections
2163 independent reflections
1363 reflections with *I* > 2σ(*I*)
*R*_int_ = 0.094

#### Refinement

*R*[*F* ^2^ > 2σ(*F* ^2^)] = 0.051
*wR*(*F* ^2^) = 0.139
*S* = 0.95
2163 reflections
155 parameters
H-atom parameters constrained
Δρ_max_ = 0.37 e Å^−3^
Δρ_min_ = −0.28 e Å^−3^

### 

Data collection: *SMART* (Bruker, 2007 ▶); cell refinement: *SAINT* (Bruker, 2007 ▶); data reduction: *SAINT*; program(s) used to solve structure: *SHELXS97* (Sheldrick, 2008 ▶); program(s) used to refine structure: *SHELXL97* (Sheldrick, 2008 ▶); molecular graphics: *SHELXTL* (Sheldrick, 2008 ▶); software used to prepare material for publication: *SHELXTL*.

## Supplementary Material

Crystal structure: contains datablock(s) I, global. DOI: 10.1107/S1600536813011598/cv5405sup1.cif

Structure factors: contains datablock(s) I. DOI: 10.1107/S1600536813011598/cv5405Isup2.hkl

Supplementary material file. DOI: 10.1107/S1600536813011598/cv5405Isup3.cml

Additional supplementary materials:  crystallographic information; 3D view; checkCIF report

## Figures and Tables

**Fig. 1.**
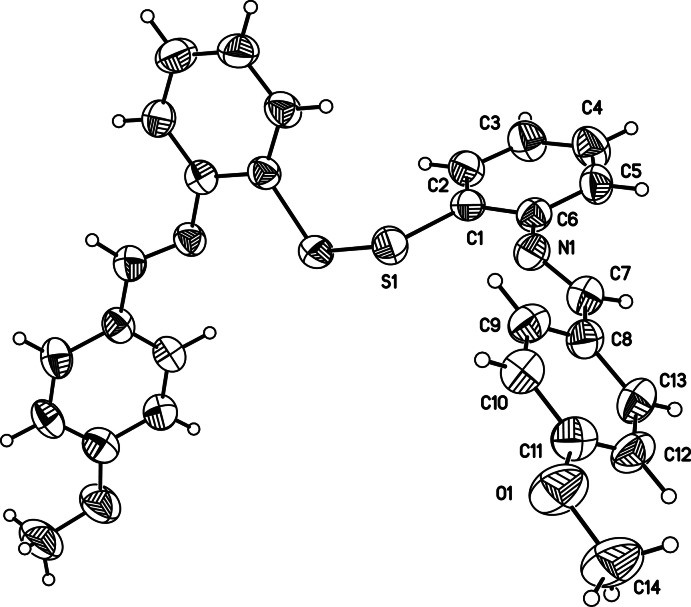
The molecular structure of (I) showing the atomic numbering and 50% probabilty displacement ellipsoids. Unlabelled atoms are related with the labelled ones by symmetry operation (-x, y, 1/2 - z).

## supplementary crystallographic information

### Comment

As a contribution to structural study of diaminodiphenyl disulfides (İde *et al.*, 1997; Wang *et al.*, 2011; He *et al.*, 2011), we present here the crystal structure of the title compound, (I).

In (I) (Fig. 1), the bond lengths and angles are normal and correspond to those observed in related compounds (İde *et al.*, 1997; Ozbey *et al.*,1998; He *et al.*, 2011). The molecule has crystallographic twofold rotation symmetry with the twofold axis passing through the midpoint of the central S—S bond. Two benzene rings connected through disulfide chain form a dihedral angle of 76.1 (1)°. Two benzene rings in two benzylideneaniline fragments form the dihedral angles of 48.9 (1)°. The crystal packing exhibits no significantly short intermolecular contacts.

### Experimental

4-Methoxybenzaldehyde (2 mmol) in ethanol (10 ml) was added to a solution of 2,2'-diaminodiphenyl disulfide (1 mmol) in ethanol (20 ml). The solution was heated to 323 K for 4 h. The reaction mixture was cooled to room tempertature and the yellow crystalline product was obtained.

### Refinement

All H atoms were placed in geometrically idealized positions (C—H 0.93–0.96 Å) and treated as riding on their parent atoms, with *U*_iso_(H) = 1.2–1.5*U*_eq_(C).

### Crystal data

**Table:**

C_28_H_24_N_2_O_2_S_2_	*D*_x_ = 1.308 Mg m^−^^3^
*M**_r_* = 484.61	Mo *K*α radiation, λ = 0.71073 Å
Orthorhombic, *P**b**c**n*	Cell parameters from 1719 reflections
*a* = 10.2657 (11) Å	θ = 2.5–22.9°
*b* = 13.0675 (13) Å	µ = 0.25 mm^−^^1^
*c* = 18.3415 (15) Å	*T* = 298 K
*V* = 2460.5 (4) Å^3^	Block, yellow
*Z* = 4	0.26 × 0.22 × 0.17 mm
*F*(000) = 1016	

### Data collection

**Table:**

Bruker SMART APEX CCD area-etector diffractometer	2163 independent reflections
Radiation source: fine-focus sealed tube	1363 reflections with *I* > 2σ(*I*)
Graphite monochromator	*R*_int_ = 0.094
phi and ω scans	θ_max_ = 25.0°, θ_min_ = 2.5°
Absorption correction: multi-scan (*SADABS*; Sheldrick, 1996)	*h* = −8→12
*T*_min_ = 0.939, *T*_max_ = 0.960	*k* = −12→15
9487 measured reflections	*l* = −17→21

### Refinement

**Table:**

Refinement on *F*^2^	Primary atom site location: structure-invariant direct methods
Least-squares matrix: full	Secondary atom site location: difference Fourier map
*R*[*F*^2^ > 2σ(*F*^2^)] = 0.051	Hydrogen site location: inferred from neighbouring sites
*wR*(*F*^2^) = 0.139	H-atom parameters constrained
*S* = 0.95	*w* = 1/[σ^2^(*F*_o_^2^) + (0.075*P*)^2^] where *P* = (*F*_o_^2^ + 2*F*_c_^2^)/3
2163 reflections	(Δ/σ)_max_ < 0.001
155 parameters	Δρ_max_ = 0.37 e Å^−^^3^
0 restraints	Δρ_min_ = −0.28 e Å^−^^3^

### Special details

**Table:**

Geometry. All e.s.d.'s (except the e.s.d. in the dihedral angle between two l.s. planes) are estimated using the full covariance matrix. The cell e.s.d.'s are taken into account individually in the estimation of e.s.d.'s in distances, angles and torsion angles; correlations between e.s.d.'s in cell parameters are only used when they are defined by crystal symmetry. An approximate (isotropic) treatment of cell e.s.d.'s is used for estimating e.s.d.'s involving l.s. planes.
Refinement. Refinement of *F*^2^ against ALL reflections. The weighted *R*-factor *wR* and goodness of fit *S* are based on *F*^2^, conventional *R*-factors *R* are based on *F*, with *F* set to zero for negative *F*^2^. The threshold expression of *F*^2^ > σ(*F*^2^) is used only for calculating *R*-factors(gt) *etc*. and is not relevant to the choice of reflections for refinement. *R*-factors based on *F*^2^ are statistically about twice as large as those based on *F*, and *R*- factors based on ALL data will be even larger.

### Fractional atomic coordinates and isotropic or equivalent isotropic displacement parameters (Å^2^)

**Table:**

	*x*	*y*	*z*	*U*_iso_*/*U*_eq_	
S1	0.01987 (8)	0.02789 (6)	0.19518 (4)	0.0510 (3)	
N1	0.1694 (2)	0.02278 (17)	0.06606 (11)	0.0433 (6)	
O1	0.0529 (2)	0.39678 (18)	−0.14776 (12)	0.0757 (7)	
C1	0.1483 (3)	−0.0635 (2)	0.18144 (14)	0.0408 (7)	
C2	0.1829 (3)	−0.1399 (2)	0.23057 (15)	0.0494 (8)	
H2	0.1390	−0.1459	0.2747	0.059*	
C3	0.2830 (3)	−0.2067 (2)	0.21360 (16)	0.0574 (8)	
H3	0.3062	−0.2575	0.2467	0.069*	
C4	0.3491 (3)	−0.1991 (2)	0.14808 (16)	0.0596 (9)	
H4	0.4153	−0.2450	0.1369	0.072*	
C5	0.3159 (3)	−0.1225 (2)	0.09907 (15)	0.0510 (8)	
H5	0.3605	−0.1171	0.0551	0.061*	
C6	0.2160 (3)	−0.0532 (2)	0.11509 (14)	0.0417 (7)	
C7	0.2471 (3)	0.0735 (2)	0.02534 (14)	0.0468 (7)	
H7	0.3359	0.0594	0.0276	0.056*	
C8	0.2011 (3)	0.1530 (2)	−0.02483 (13)	0.0451 (7)	
C9	0.0684 (3)	0.1658 (2)	−0.03945 (14)	0.0483 (7)	
H9	0.0089	0.1192	−0.0205	0.058*	
C10	0.0241 (3)	0.2456 (2)	−0.08118 (15)	0.0518 (8)	
H10	−0.0646	0.2524	−0.0902	0.062*	
C11	0.1108 (3)	0.3167 (2)	−0.11021 (14)	0.0518 (8)	
C12	0.2432 (3)	0.3044 (2)	−0.09884 (14)	0.0541 (8)	
H12	0.3024	0.3505	−0.1187	0.065*	
C13	0.2868 (3)	0.2216 (2)	−0.05696 (14)	0.0539 (8)	
H13	0.3758	0.2123	−0.0505	0.065*	
C14	0.1355 (4)	0.4714 (3)	−0.1798 (2)	0.0852 (12)	
H14A	0.1930	0.4987	−0.1434	0.128*	
H14B	0.0836	0.5257	−0.1998	0.128*	
H14C	0.1859	0.4404	−0.2180	0.128*	

### Atomic displacement parameters (Å^2^)

**Table:**

	*U*^11^	*U*^22^	*U*^33^	*U*^12^	*U*^13^	*U*^23^
S1	0.0584 (6)	0.0438 (5)	0.0509 (5)	0.0064 (4)	0.0120 (4)	0.0093 (3)
N1	0.0474 (15)	0.0441 (13)	0.0383 (12)	−0.0016 (11)	0.0033 (11)	0.0047 (11)
O1	0.0769 (17)	0.0664 (16)	0.0839 (16)	−0.0040 (13)	−0.0048 (13)	0.0296 (13)
C1	0.0446 (18)	0.0362 (15)	0.0415 (15)	−0.0036 (13)	0.0016 (13)	−0.0014 (12)
C2	0.064 (2)	0.0450 (18)	0.0395 (15)	−0.0004 (15)	0.0080 (14)	0.0022 (13)
C3	0.069 (2)	0.0495 (19)	0.0536 (18)	0.0127 (17)	0.0051 (16)	0.0100 (15)
C4	0.064 (2)	0.054 (2)	0.061 (2)	0.0174 (16)	0.0045 (16)	0.0008 (16)
C5	0.054 (2)	0.0534 (19)	0.0451 (16)	0.0032 (16)	0.0130 (14)	−0.0001 (14)
C6	0.0428 (17)	0.0428 (16)	0.0395 (15)	−0.0035 (13)	−0.0009 (13)	−0.0001 (12)
C7	0.0447 (18)	0.0508 (17)	0.0449 (15)	−0.0015 (15)	0.0022 (14)	0.0015 (13)
C8	0.053 (2)	0.0492 (17)	0.0330 (14)	−0.0047 (14)	0.0048 (13)	−0.0003 (12)
C9	0.0509 (19)	0.0487 (18)	0.0452 (16)	−0.0089 (15)	0.0002 (14)	0.0031 (13)
C10	0.0499 (19)	0.0558 (19)	0.0496 (17)	−0.0019 (16)	−0.0017 (14)	0.0028 (15)
C11	0.063 (2)	0.0533 (19)	0.0389 (15)	−0.0033 (17)	−0.0038 (15)	0.0040 (14)
C12	0.065 (2)	0.0540 (19)	0.0436 (16)	−0.0157 (17)	0.0081 (15)	0.0101 (14)
C13	0.0511 (19)	0.066 (2)	0.0444 (16)	−0.0081 (16)	0.0066 (14)	0.0069 (15)
C14	0.101 (3)	0.065 (3)	0.090 (3)	−0.011 (2)	0.005 (2)	0.030 (2)

### Geometric parameters (Å, º)

**Table:**

S1—C1	1.797 (3)	C7—C8	1.466 (4)
S1—S1^i^	2.0518 (14)	C7—H7	0.9300
N1—C7	1.278 (3)	C8—C13	1.388 (4)
N1—C6	1.423 (3)	C8—C9	1.398 (4)
O1—C11	1.387 (4)	C9—C10	1.372 (4)
O1—C14	1.420 (4)	C9—H9	0.9300
C1—C2	1.390 (4)	C10—C11	1.392 (4)
C1—C6	1.408 (3)	C10—H10	0.9300
C2—C3	1.385 (4)	C11—C12	1.385 (4)
C2—H2	0.9300	C12—C13	1.400 (4)
C3—C4	1.383 (4)	C12—H12	0.9300
C3—H3	0.9300	C13—H13	0.9300
C4—C5	1.388 (4)	C14—H14A	0.9600
C4—H4	0.9300	C14—H14B	0.9600
C5—C6	1.398 (4)	C14—H14C	0.9600
C5—H5	0.9300		
			
C1—S1—S1^i^	106.46 (9)	C13—C8—C9	117.3 (3)
C7—N1—C6	121.4 (2)	C13—C8—C7	121.3 (3)
C11—O1—C14	117.9 (3)	C9—C8—C7	121.3 (3)
C2—C1—C6	120.1 (3)	C10—C9—C8	121.4 (3)
C2—C1—S1	125.0 (2)	C10—C9—H9	119.3
C6—C1—S1	114.8 (2)	C8—C9—H9	119.3
C3—C2—C1	119.8 (3)	C9—C10—C11	120.6 (3)
C3—C2—H2	120.1	C9—C10—H10	119.7
C1—C2—H2	120.1	C11—C10—H10	119.7
C4—C3—C2	120.9 (3)	C12—C11—O1	125.7 (3)
C4—C3—H3	119.6	C12—C11—C10	119.5 (3)
C2—C3—H3	119.6	O1—C11—C10	114.8 (3)
C3—C4—C5	119.7 (3)	C11—C12—C13	119.1 (3)
C3—C4—H4	120.2	C11—C12—H12	120.5
C5—C4—H4	120.2	C13—C12—H12	120.5
C4—C5—C6	120.7 (3)	C8—C13—C12	122.0 (3)
C4—C5—H5	119.7	C8—C13—H13	119.0
C6—C5—H5	119.7	C12—C13—H13	119.0
C5—C6—C1	118.8 (2)	O1—C14—H14A	109.5
C5—C6—N1	124.4 (2)	O1—C14—H14B	109.5
C1—C6—N1	116.5 (2)	H14A—C14—H14B	109.5
N1—C7—C8	122.2 (3)	O1—C14—H14C	109.5
N1—C7—H7	118.9	H14A—C14—H14C	109.5
C8—C7—H7	118.9	H14B—C14—H14C	109.5

Symmetry code: (i) −*x*, *y*, −*z*+1/2.
